# Osteochondritis dissecans located on the medial tibial plateau: a case report

**DOI:** 10.1186/s13256-016-1164-4

**Published:** 2017-01-04

**Authors:** Yimin Zhang, Xiaoguang Liu

**Affiliations:** Weifang People’s Hospital, Weifang, China

**Keywords:** Osteochondritis dissecans, Knee, Tibial plateau

## Abstract

**Background:**

Osteochondritis dissecans are typically located mainly on the femoral condyle, with reported but less common cases of patella involvement. In this case report, we reported a rare case of osteochondritis dissecans located on the medial tibial plateau.

**Case presentation:**

A 26-year-old Han Chinese woman presented with a lesion on her left medial tibial plateau. She was treated by arthroscopy for her knee condition. During the operation, a primary diagnosis of osteochondritis dissecans on her medial tibial plateau was indicated by an osteochondral fragment. Arthroscopic removal of the fragment was then performed. Histological examination which was done by a pathologist after the operation supported the positive pathology of osteochondritis dissecans.

**Conclusions:**

The common site of osteochondritis dissecans development in the knee is on the femoral condyle, and some develop on the patella. In this case report, it was proven that osteochondritis dissecans can also take place on the tibial plateau, although it is very uncommon.

## Background

The term osteochondritis dissecans (OCD) was first coined in 1887 by König [1], who suggested that this pathology was mainly due to inflammation, not trauma. But, at present, OCD is regarded as an acquired lesion of subchondral bone characterized by degrees of osseous resorption, collapse, and sequestrum formation with possible involvement of the articular cartilage through delamination without acute osteochondral fracture. Thus, many causes, such as trauma, inflammation, genetics, vascular abnormalities, and constitutional factors [2] were postulated for the etiologies responsible for OCD. The exact pathophysiology, natural history, and treatment remain unclear, but some advancement has been made in our understanding of OCD [3].

Common sites for OCD of the knee include the lateral aspect of the medial femoral condyle, the central portion of the medial femoral condyle, the lateral femoral condyle, the medial aspect of the medial femoral condyle, and the patella [4]. In this case report we reported a case of OCD on the medial tibial plateau, which is far more uncommon.

## Case presentation

**Fig. 1:**
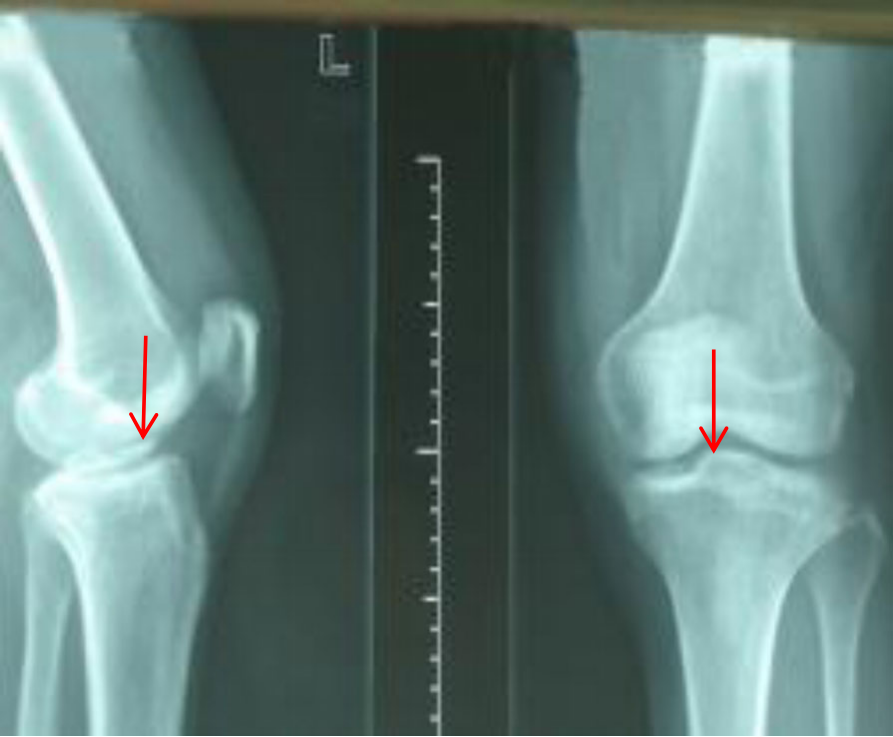
X-ray image showing an osteochondral fragment on the tibial plateau that may be confused for an avulsion fracture of the tibial insertion of the anterior cruciate ligament (*red arrow*)

**Fig. 2:**
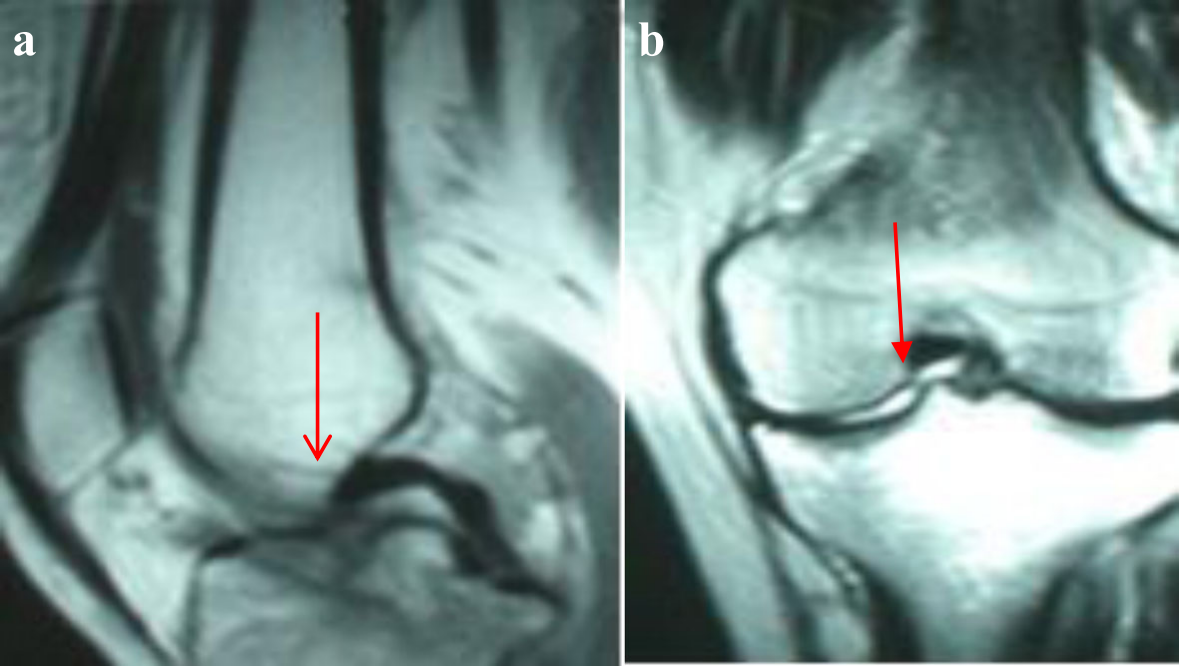
The Osteochondral fragment and its location on medial tibial plateau in magnetic resonance images. (**a**) *Red arrow* revealed the osteochondral fragment and its location on medial tibial plateau in sagittal magnetic resonance images. (**b**) *Red arrow* revealed the osteochondral fragment and its location on medial tibial plateau in coronal magnetic resonance images

A 26-year-old Han Chinese woman presented with a lesion on her left medial tibial plateau. She was an office employee with no athletic history; she denied any injury or symptom before she experienced a sudden sharp pain and instability of the affected knee while going downstairs. Her symptoms did not spontaneously relieve after 2 days of rest so she presented to our hospital. A physical examination revealed a swollen knee joint with Floating patellar test (+), Anterior drawer test (−), Posterior drawer test (−), Lachman test (−), Abduction stress test (−), and Adduction stress test (−). A McMurray sign could not be done accurately due to pain and swelling in her knee from 20° of extension to 120° of flexion. She had no neurological symptoms and no other physical signs were apparent. Of interest, a mistaken diagnosis of a lower insertion avulsion fracture of anterior cruciate ligament could be made at first glance at X-ray and magnetic resonance imaging (MRI) (Figs. 1 and 2).

**Fig. 3:**
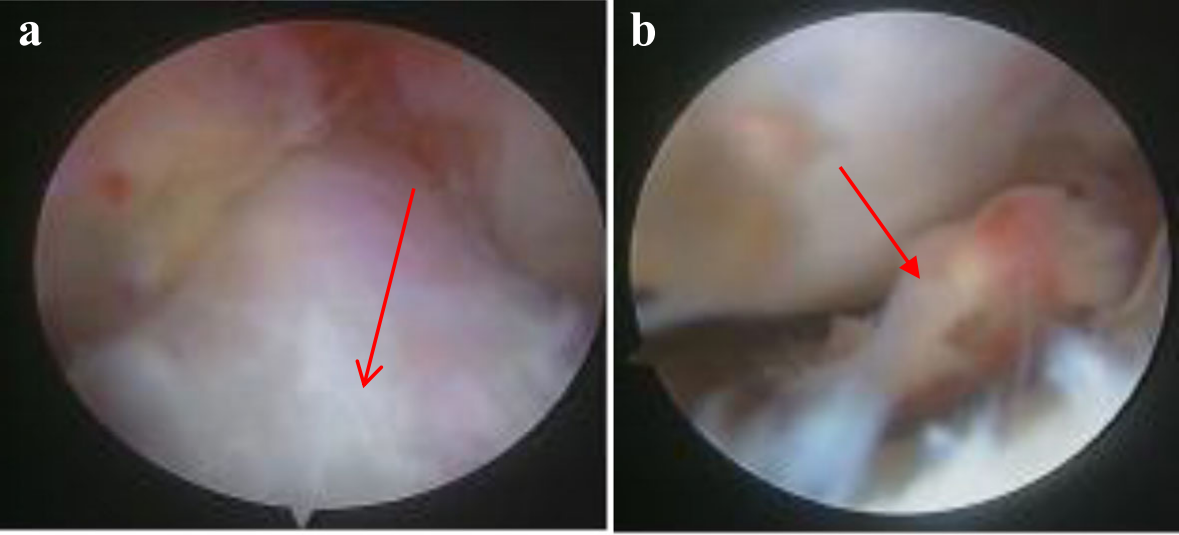
The normal distal anterior cruciate ligament insertion and the osteochondral fragment on the medial tibial platear during arthroscopy. (**a**) *Red arrow* revealed the normal distal anterior cruciate ligament insertion. (**b**) *Red arrow* revealed the osteochondral fragment on the medial tibial plateau near the insertion as seen during arthroscopy

She was treated with arthroscopy (Fig. 3). During the operation, a primary diagnosis of OCD on her medial tibial plateau was indicated by an osteochondral fragment near the lower insertion of her anterior cruciate ligament, and the fragment was removed arthroscopically. Microfracture of the defect area was then performed. Histological examination which was done by a pathologist after the operation supported the positive pathology of OCD.

Postoperatively, weight-bearing was disallowed for the repair of the osteochondral defect where the microfracture was done, whereas quadriceps muscle training, such as straight leg raising, was encouraged. Continuous passive motion was also used for extension and flexion of her knee for 3 weeks after her operation. Three weeks later, rehabilitation began from partial weight-bearing up to total weight-bearing with no effusion of her knee joint. At 3 months of follow-up, she could walk and ride a bicycle freely except for a little pain after lengthy activity, particularly when going upstairs and downstairs. She was asked to avoid competitive activities for at least half a year or even for all her life, but muscle training was necessary; we suggested some helpful sports such as swimming. At 6 months, she was told that she could engage in long walks and even jogging with full knee flexion and extension.

## Discussion

OCD is a well-recognized yet poorly understood condition. The etiology and treatment of this pathology still remain controversial. On many occasions, the symptoms of OCD are not very clear, leading to a delayed or difficult diagnosis. Radiography may be helpful as an X-ray is reliable for a diagnosis and MRI can be obtained for further characterization of the lesion [5].

When considering an OCD diagnosis, the presentation, clinical signs, and a radiographic examination are indispensable. Once the diagnosis has been established, treatment strategies such as conservative therapy, arthroscopic drilling [6], screw fixation, bone grafting, fixation with autograft osteochondral plugs, and salvage procedures (autologous chondrocyte implantation and fresh osteochondral allografts) should be considered [4].

In this report, we briefly examined the evolution of our understanding of OCDs from Professor König’s first description in 1887 until the present. The diagnosis of an OCD was mainly dependent on imaging, such as an X-ray or MRI; the location of the OCD could also be fully characterized.

The most common site of OCD development in the knee is the lateral aspect of the medial femoral condyle, which occurs in 51% of cases according to Hefti *et al*. [4]. In their multicenter series of 509 knees in 452 patients, the authors found other sites that were involved less frequently: the central medial femoral condyle (19%), the lateral femoral condyle (17%), the medial side of the medial femoral condyle (7%), and the patella (7%). Irregular ossification centers of the distal femoral condyle tend to be found more posterior on the condyle (although they can be anywhere on the condyle) and are associated with patients of a younger age. In the literature, some authors have reported that OCD can occur on the lateral tibial plateau [7], and we supplemented occurrences of OCD located on the medial tibial plateau through this case. So, the pathogenesis of such a lesion should be discussed in future studies.

## Conclusions

The common site of OCD development in the knee is on the femoral condyle, and some develop on the patella. In this case report, it is proven that OCD can also take place on the tibial plateau, although it is very uncommon.

## Abbreviation

OCD: Osteochondritis dissecans
